# Morals of Sickness

**Published:** 1859-11

**Authors:** 


### MORALS OF SICKNESS.

There are certain forms of disease which, while they waste the body, depress the mind, and stupify the moral sentiment; hence, the wise physician often feels compelled to address his remedies to the mind, to bring the religious element into requisition, in strong appeals to a sense of duty. Sometimes there is not left energy enough for an effort at restoration. This is often the case with clergymen, literary men, and professors in colleges. One of these is like a man just entering the current above the falls of Niagara; he is sensible of his danger, feels that in a short time all effort will be unavailing, yet he has not the moral energy requisite to make use of the means necessary for his deliverance. This condition is in nearly all cases the result of *dyspepsia*, that is, it is the result of a want of thorough digestion of the food, a defect which is brought on by injudicious eating. Persons who use opium, tobacco, liquors, or strong coffee and tea, eventually fall into this same state. No Christian man will have any difficulty in saying that the use of liquors should be given up as a duty, under such circumstances. But let the physician of acknowledged science and ability press upon that same man the duty of abandoning the use of tobacco, or of adopting a plainer mode of feeding, he will find his appeals powerless. Can a man be guiltless who condemns his neighbour for drinking errors, but does not condemn himself for errors in eating? In other cases, where comparatively little is needed beyond a pill or two a month for a short time, except judicious exercise, the prescription is met with, "Well, I cannot spare the time, my professional duties are such that I have not the leisure." But suppose you die, what then? You cannot lose *now* an hour a day, *then* ALL time is lost!

Physicians well know that three-fourths of the ordinary attacks of sickness are the result of imprudence; that if men lived wisely, the average age would be full three score years and ten, instead of half that term, as it now is.

We know that if human life is valuable to all, the increase of its duration would increase its value. That if any man is useful to the church or the world, from thirty to forty, he would be still more useful from fifty to sixty; and that it is his duty to protract his usefulness, there can be no doubt.

Again, none will deny that a man in robust health is more available in any calling than he would be if he were an invalid. If then it is the duty of every one to do the largest amount of good possible for him to do, he is doing a wrong to society, and to his master in heaven, if he fail to use the means to avoid disease, and to keep him in robust health; that is, if he fail to inform himself as to the best method of accomplishing such results.

The most terrible of all spiritual conditions, as well as the most utterly hopeless, is for a man to be conscious of his going to perdition, and yet to feel a total indifference to his situation, so much so, as to be incapable of making any effort to escape the ruin. Such is the bodily condition of many persons; they are not sufficiently alive to their situation to be stimulated to proper efforts for their deliverance, by any appeals to duty, whose end is death!

